# Conditional DNA repair mutants enable highly precise genome engineering

**DOI:** 10.1093/nar/gku105

**Published:** 2014-02-05

**Authors:** Ákos Nyerges, Bálint Csörgő, István Nagy, Dóra Latinovics, Béla Szamecz, György Pósfai, Csaba Pál

**Affiliations:** ^1^Synthetic and Systems Biology Unit, Institute of Biochemistry, Biological Research Centre of the Hungarian Academy of Sciences, Szeged H-6726, Hungary and ^2^Symbiosis and Functional Genomics Unit, Institute of Biochemistry, Biological Research Centre of the Hungarian Academy of Sciences, Szeged H-6726, Hungary

## Abstract

Oligonucleotide-mediated multiplex genome engineering is an important tool for bacterial genome editing. The efficient application of this technique requires the inactivation of the endogenous methyl-directed mismatch repair system that in turn leads to a drastically elevated genomic mutation rate and the consequent accumulation of undesired off-target mutations. Here, we present a novel strategy for mismatch repair evasion using temperature-sensitive DNA repair mutants and temporal inactivation of the mismatch repair protein complex in *Escherichia coli*. Our method relies on the transient suppression of DNA repair during mismatch carrying oligonucleotide integration. Using temperature-sensitive control of methyl-directed mismatch repair protein activity during multiplex genome engineering, we reduced the number of off-target mutations by 85%, concurrently maintaining highly efficient and unbiased allelic replacement.

## INTRODUCTION

Recent breakthroughs in genome engineering have greatly expanded our ability to design organisms in a directed and combinatorial manner. The myriad of novel genome-scale modification technologies offer new opportunities for the construction of biological systems with desired properties (1). From these techniques, oligonucleotide (oligo)- mediated allelic replacement has been optimized toward multiplexing and automation (2). Specifically, multiplex-automated genome engineering (MAGE) is capable of editing and evolving the genome of a desired organism. Several factors make MAGE exceptionally attractive for cell manipulation. It enables simultaneous and cost-efficient modification of a large set of genomic targets and accordingly, exploration of the effects of mutations in a combinatorial manner by generating a diverse heterogenic population across the targeted loci. The power of MAGE has been demonstrated in a wide range of biotechnological applications. It allows (i) optimization of metabolic pathways to produce industrially relevant compounds (2,3), (ii) improvement of bacterial growth properties under selected conditions (4) and (iii) genome-wide replacement of a specific codon in *Escherichia coli* (5,6). Therefore, MAGE has the potential to transform basic research by accelerating and expanding the range of protocols for genome editing and analysis (1).

MAGE relies on the incorporation of synthetic single-stranded DNA (ssDNA) oligonucleotides carrying the desired modifications into the lagging strand of the replicating target genome (7,8). The efficiency of this process is highly dependent on the avoidance of the methyl-directed mismatch repair (MMR) machinery of the target cell. The removal of the endogenous MMR system increases the rate of oligo incorporation by orders of magnitude and eliminates biases in the rate of incorporation of different types of mismatches (9). However, the need for a disabled MMR machinery also presents the greatest drawback: global MMR inactivation results in an ∼100-fold increase in host mutation rate (10) leading to the accumulation of unwanted background mutations across the bacterial genome. For example, in an effort to replace all TAG stop codons in *E. coli*, three different strains were sequenced after 25–30 cycles of MAGE and subsequent genome transfer. All of them were found to carry >100 off-target mutations (5). The severity of this problem is evident: these off-target mutations can potentially mask the phenotypic effects of the engineered modifications. Several previous approaches have been proposed to solve this problem (9,11–13) (Table 1), but all suffer from serious limitations, are technically challenging or introduce novel methodological problems.

**Table 1.** Various approaches to achieve high allele replacement (AR) efficiency while minimizing mutator phenotype

General strategy	Specific attempts	Possible limitations
Modifications in the ssDNA oligo	C:C mismatch in vicinity of target modification (11)	Introduce novel scars, place sequence limitations on which genome modifications can be made, problems involving codon bias (11)
	Change of wobble position of two neighboring codons of target modification (11)	
	Use of chemically modified analogs of the four base pairs (12)	Added cost and lesser availability of the modified bases, possible toxic effects on the host organism (12)

Diversion of MMR proteins	Use of adenine analog 2-aminopurine to titrate MutL protein (9)	10-fold less efficient than with MMR deficient strains, extra incubation time, mutagenic effect of 2-aminopurine (9)
	Co-electroportation of dsDNA oligo containing mismatches (9)	No effect on AR efficiencies even when present in 100-fold excess (9)

Control of MMR protein transcription	Reversible inactivation of MMR protein coding gene using oligonucleotide (13)	Requires extra MAGE cycles for the enrichment of the introduced modification, mutator phenotype persists during entire cycles when the gene is turned off (13)

The elimination of MMR activity is only necessary during a relatively brief period of each MAGE cycle (i.e. during allelic replacement). Therefore, we suggest a simple strategy relying on temperature-based control of MMR protein activity. In contrast to the relatively slow process of transcriptional control of MMR protein level, our protocol applies MMR protein variants with temperature-sensitive defects, allowing rapid switching between mutator and nonmutator states; thus, minimizing the time the bacterial population spends susceptible to the accumulation of off-target mutations. This temporal on–off switch of mismatch repair is easily incorporated into the standard MAGE protocol, as it already uses a temperature shift to activate the expression of the λ Red recombinase enzymes.

## MATERIALS AND METHODS

### Media, chemicals and reagents

Unless otherwise noted, cultures were grown in Luria-Bertani-Lennox (LB^L^) media (10 g of tryptone, 5 g of yeast extract, 5 g of sodium chloride per 1 L of water) for cell manipulations. For the isolation of single colonies for further analysis, cells were sampled on LB^L^ media supplemented with 1.5% agar and tetracycline at a working concentration of 20 µg/ml. For the measurement of *rpsL* allelic replacement frequency, LB^L^ agar was supplemented with 50 µg/ml streptomycin. LacZ, MalK and AraB activities were assayed on MacConkey agar base (peptone 20 g, bile salts 1.5 g, sodium chloride 5 g, agar 13.5 g, neutral red 0.03 g and crystal violet 1.0 mg per 1 L of water) supplemented with 20 µg/ml tetracycline and 1% of lactose for LacZ, maltose for MalK or l-arabinose for AraB activity measurements Glycerol-free terrific broth (TB) was applied for recovery media (yeast extract 24 g, tryptone 12 g, K_2_HPO_4_ 9.4 g, KH_2_PO_4_ 2 g per 1 L of water).

### Oligonucleotides

All oligonucleotides for allelic replacement as well as polymerase chain reaction (PCR) primers used in this study are presented in Supplementary File S1. Oligos were ordered with standard purification and desalting from Integrated DNA Technologies. Oligos applied for allelic replacement have complementary sequence to the replicating lagging strand and have minimized secondary structure (>−12 kcal mol^−^^1^). Additionally, each oligo contained two subsequent phosphorothioate linkages at both 5′ and 3′ termini for endogenous nuclease evasion.

### Strain construction

All applied strains were derived from *E. coli* K-12 MG1655. For the construction of temperature-sensitive mismatch repair deficient strain *E. coli* tMMR, previously described temperature-dependent *mutS*(A134V) and *mutL*(G62S) alleles (14) were introduced into wild-type background.

Single-nucleotide variation in mutS, which confers an Ala134→Val amino acid change, was constructed by using a suicide plasmid-based method. Standard steps and plasmids (pST76A, pSTKST) of the procedure have been described (15). Briefly, using PCR primers carrying the desired point mutation (Supplementary File S1), the mutant *mutS* gene was constructed, then cloned into the thermosensitive pST76A plasmid. The plasmid construct was then transformed into the cell, where it was able to integrate into the chromosome by way of a single crossover between the mutant allele and the corresponding chromosomal region. The desired cointegrates were selected using the antibiotic resistance carried on the plasmid at a nonpermissive temperature for plasmid replication. Next, the pSTKST helper plasmid was transformed, then induced within the cells, resulting in the expression of the I*Sce*-I meganuclease enzyme, which cleaves the chromosome at the 18-bp recognition site present in the integrated plasmid. The resulting chromosomal break is repaired by way of RecA-mediated intramolecular recombination between the homologous segments in the vicinity of the broken ends. The recombinational repair results in either a reversion to the wild-type chromosome or in a scarless allele replacement. Strains carrying the desired point mutation were distinguished by sequencing the given chromosomal region, resulting in the MG1655 tMutS strain. Whole-genome sequencing of the constructed strain revealed a synonymous undetected point mutation in the mutS gene (1323C→T), which does not result in amino acid change and has no effect on the desired phenotype.

Oligonucleotide-mediated singleplex λ Red recombination was used to generate the temperature-dependent mutL(G62S) variant Gly62→Ser in MG1655 tMutS. To generate the mutL point mutation, a plasmid-based iterative λ Red recombination protocol was used. As the integrated mismatch (G:T chromosomal-to-synthetic) has high correction efficiency without MMR inactivation, the allelic replacement was done at elevated temperature to partially inactivate MutS proteins during oligo incorporation (experiment based on unpublished results). The temperature-sensitive *mutS* mutant strain, MG1655 tMutS, carrying the pBADαβγ λ Red expression plasmid (16) was applied. In brief, to perform allelic replacement, cells were grown in 10-ml LB^L^ from overnight starter culture at 38°C, 250 rpm to OD_550_ 0.5–0.7. λ Red proteins were induced by the addition of l-arabinose at 0.2% concentration for 30 min. For recombination, cells were pelleted 3800 rpm for 7 min and washed twice in ice-cold purified water (dH_2_O), resuspended in dH_2_O and electroporated with oligo MutL35_SNP at 2.5 µM final concentration. Electroporated cells were allowed to recover in 10 ml of LB at 38°C until the culture reached mid-logarithmic growth. After two iterations, cells were plated on LB^L^ agar plates. Clones with desired mutation were identified by allele-specific PCR.

Briefly, selected colonies were assayed in 25-µl colony PCR reactions using DreamTaq DNA Polymerase (Thermo Scientific, catalog number EP0702) in DreamTaq Buffer (includes 2 mM MgCl_2_) and 200 µM deoxyribonucleotide triphosphates (Thermo Scientific, catalog number R0192), 0.2 µM MutL35ASP_fw and _rv primers and 0.5 µl of saturated bacterial culture. For allele discrimination, the following PCR protocol was used: heat inactivation and cell lysis at 96°C for 5 min, 30 cycles (95°C for 30 s, 63.5°C for 30 s and 72°C for 40 s) and final extension for 5 min at 72°C. Colonies containing the desired point mutation was confirmed by sequencing the target site in *mutL*. PCR amplicons, generated by the MutLseq_fw and MutL35ASP_rv primers in colony PCR, were sequenced using the MutLseq_fw primer for sequencing.

Permanent mismatch repair deactivation was achieved by endogenous *mutS* gene removal by suicide plasmid-based scarless deletion and yielded *E. coli* K-12 MG1655 Δ*mutS*.

The heat shock-inducible genomic λ Red system was P1 transduced from *E. coli* LT521 {MG1655 gal490 *nadA*::Tn10 pglΔ8 [λ cI857 Δ(*cro-bioA*)]} (courtesy of Donald L. Court, National Cancer Institute, Frederick, MD, USA). This λ prophage-based construct was integrated at the *bioA/bioB* locus of the constructed strains and allowed the induction of *gam, beta* and *exo* genes by a brief heat shock at 42°C. Integration of the prophage construct into *E. coli* tMMR yielded MGλ-tMMR. MGλ-Δ*mutS* was created by prophage introduction into *E. coli* K-12 MG1655 Δ*mutS*.

### MAGE cycling process

Individual colonies from freshly streaked overnight agar plates were inoculated into glass flasks containing 10-ml LB^L^ aliquots supplemented with 20 µg/ml tetracycline and incubated in a shaking incubator at 250 rpm at 32°C. On reaching OD_550_ 0.55–0.65, flasks were moved to a prewarmed 42°C shaking water bath for 15 min 300 rpm to induce expression of λ *gam, beta* and *exo* recombinase genes. Induced cells were then immediately chilled on ice with vigorous shaking for at least 5 min. Cells were made electrocompetent by washing and pelleting twice in 10 ml of ice-cold dH_2_O at 3800 rpm for 7 min in an Eppendorf 5702 *R* centrifuge with swing-out rotor at 4°C. Finally, cells were suspended in 160 µl of dH_2_O. All cell manipulations were performed on ice.

For electroporation, 40 µl of competent cells were mixed with 1 µl of 100 µM oligos suspended in TE buffer for singleplex allelic replacement to reach 2.5-µM ssDNA concentration. For six-plex allelic replacements, 6 µl of oligo mixture was added, containing 1 µl of each oligo at ∼2.5 mM final concentration. Cells were subjected to electroporation in a prechilled 1-mm gap VWR Signature Electroporation cuvette (VWR, catalog number 89047–206) using a BioRad MicroPulser electroporator using the following parameters: 1800 V, 25 µF, 200 Ω. Five milliliters of prewarmed (36°C) TB medium was immediately added to the electroporated cells and was transferred to culture flasks. Cells were allowed to recover for 60 min either at 32 or 36°C, depending on the experiment and the applied strain. Five milliliters room temperature LB^L^ medium supplemented with 20 µg/ml tetracycline was then added, and cells were allowed to grow at 32°C until mid-logarithmic state at 250 rpm. At this point, cells were either subjected to additional MAGE cycles or assayed for phenotype and genotype analysis. Overall, three separate incubators were used during each cycle, each preset to the given temperature, allowing for immediate shifts in the temperature. The above MAGE cycling protocol was applied generally, except when noted in the experiment description.

### Optimization of recovery time

To determine the optimal time interval that cells spend at restrictive temperature during each MAGE cycle, a single allelic replacement was performed using LacZ_m10_v2lo oligo. This oligo introduces a nonsense mutation by two consecutive mismatches into the *lacZ* gene and has high correction efficiency in the presence of the native MMR system. For the measurement of the correlation between the time interval that cells spend at restrictive temperature and allelic replacement efficiency after electroporation, LacZ_m10_v2lo oligo were introduced into MGλ-tMMR in 2.5-µM final concentration. After electroporation, cells were resuspended in 15-ml prewarmed TB broth and incubated at 36°C. One milliliter of aliquots was removed and placed into 32°C shaking incubator every 15 min for overnight incubation at 250 rpm in glass tubes. Allelic replacement efficiencies were determined by plating cells at appropriate dilution from overnight culture to MacConkey media supplemented with 1% of lactose.

### Analyzing recombination

For the analysis of allelic replacement frequencies during MAGE cycles, two types of selection marker genes were selected for targeting, either to introduce nonsense or missense mutations. The first set of genes, including *lacZ*, *malK*, *araB* and *rpsL*, were selected for easy visible discrimination of recombinant colonies on supplemented MacConkey agar plates, based on sugar fermentation and/or antibiotic resistance (*rpsL*, which confers resistance to streptomycin). The second set of genes, consisting of *cycA* and *hisB*, were targeted by knock-out oligonucleotides to introduce nonsense mutations. Recombinant colonies from recombineering with the second gene set were screened by using allele-specific PCR for allele discrimination.

Targeted inactivation of LacZ activity, by the incorporation of a nonsense mutation, was assayed by plating cells onto MacConkey agar supplemented with 1% lactose as a carbon source and 20 µg/ml tetracycline. Cells were plated at appropriate dilutions in three replicates, typically to yield 400–800 colonies per plate. Plates were incubated at 30°C overnight. Allelic replacement efficiencies were calculated by dividing the number of white (LacZ-) recombinant colonies by the number of total colonies. For assaying MalK and AraB activities, the same protocol was applied with the exception of the use of MacConkey media supplemented with either 1% of maltose or l-arabinose, respectively.

*RpsL* allelic replacement frequency was measured by plating cells in parallel onto LB^L^ agar and LB^L^ agar supplemented with 50 µg/ml streptomycin. *RpsL* allelic replacement efficiencies were calculated by dividing the number of Str^R^ colonies by the number of total colonies on LB^L^ agar plates.

As it has been reported previously, *E. coli* cells can contain up to eight partial chromosomal copies during exponential growth in rich media (17). Fast growing cells that have not undergone enough cell divisions to segregate mutant and wild-type alleles, therefore, generate sectored colonies. To overcome this scenario, cells were allowed to grow overnight to early stationary phase before plating.

### Allele-specific colony PCR

Allele-specific colony PCR was used to genotype clones to measure allelic replacement frequencies for *cycA* and *hisB* and to select for clones with desired numbers of allelic replacements. For rapid allele discrimination, two sets of primers were synthesized either targeting *cycA* or *hisB*. For each set, one primer carried the corresponding mismatch at the 3′ terminus, whereas the other had complete homology to the target region. Only a clone containing the mutant allele generated a PCR product. To avoid nonspecific product formation during PCR, the optimal annealing temperature and cycling protocol was determined by gradient PCR with annealing temperatures varied between 58 and 70°C.

The query strains were assayed in 25 µl of PCR reactions using DreamTaq DNA Polymerase (Thermo Scientific) in DreamTaq Buffer (includes 2 mM MgCl_2_) and 200 µM deoxyribonucleotide triphosphates (Thermo Scientific), 0.2 µM forward and reverse primer and 0.5 µl of saturated bacterial culture (produced either by diluting one bacterial colony in 100 µl of dH_2_O or growing cells to stationary phase in LB^L^). For *cycA* allele discrimination, the following PCR cycles were used: heat inactivation and cell lysis at 96°C for 5 min, 28 cycles (95°C for 20 s, 61°C for 30 s and 72°C for 40 s) and final extension for 5 min at 72°C.

For *hisB* alleles, the optimal PCR protocol was the same with the exception of annealing temperature, which was set to 68°C.

The PCR products were separated on 1% agarose gel and were imaged by a BioDoc-It™ BioImaging System (UVP).

### Genotype analysis

Genotype analysis at selected loci of cells that underwent recombineering was performed by three main methods, which included allele-specific colony PCR, Sanger DNA sequencing and whole-genome resequencing.

### Sanger sequencing

Genotype analysis at selected loci of cells undergoing multiplex recombineering was performed by Sanger capillary sequencing, in addition to allele-specific PCR as previously described. Genotype of regions corresponding to the annealing sites for the allelic replacement oligonucleotides and its proximity were determined in this manner. In all, 600–700-bp PCR amplicons covering the target sites of all targeted mutations were sequenced from individual colonies. Amplicons were generated by colony PCR, purified using Viogene Gel/PCR DNA Isolation System (Viogene BioTek Corp., catalog number GP1002) and sequenced on a 3500 Series Genetic Analyzer (Life Technologies; LT). Mutations were identified by aligning sequence reads to the reference *E. coli* K-12 MG1655 genome (GenBank accession number NC000913; version NC_000913.2 GI:49175990).

For the analysis of variation in the target sites of MAGE oligos, 96 mutants of both MGλ-Δ*mutS* and MGλ-tMMR strains were sequenced across the target site of the cycA-AAAC oligo within the *cycA* gene after undergoing 20 MAGE iterations each. Individual colonies from populations that had undergone MAGE cycling were plated and isolated on LB^L^ plates. Colony PCR, using the cycA1 and cycAASP_r primers, was used to amplify the oligo target site and its surroundings. The PCR amplicons were purified using ZR-96 DNA Clean-up Kit™ (Zymo Research, catalog number D4018). Capillary sequencing was performed by Macrogen Inc., Amsterdam, Netherlands, via EZ-Seq DNA sequencing service, using the cycA1 primer for sequencing. The obtained sequences were aligned to the *E. coli* K-12 substr. MG1655 chromosome using Genomics Workbench 6.0.1 (CLC Bio). Variations mapped within the target site of the cycA_AAAC oligo were judged as within-site mutations.

### Whole-genome sequencing

Genomic off-target mutation rates were determined by analyzing five 6-fold mutants from each MAGE-cycled population and comparing their genome with the sequenced ancestral strains. *LacZ*, *malK* and *araB* triple knock-out mutants harboring an *rpsL* Str^R^ mutation were selected by plating cells to MacConkey media base supplemented with 0.5% lactose, 0.5% maltose, 0.5% l-arabinose and 50 µg/ml streptomycin at 30°C. Selected colonies were then screened for harboring *cycA* and *hisB* mutations using allele-specific PCR. Cell lines were then confirmed for containing the desired targeted mutations by Sanger sequencing using primers listed in Supplementary File S1. Selected cell lines were subjected to whole-genome resequencing, and SNV and INDEL analysis was performed.

Briefly, genomic DNA was extracted from selected *E. coli* isolates (GenElute™ Bacterial Genomic DNA kit, Sigma-Aldrich, catalog number NA2110), and the subsequent library preparation was performed using the 5500 SOLiD Fragment Library Core Kit (LT). Three micrograms of purified bacterial genomic DNA was fragmented by Covaris S2 System to 100–250 bp. The fragmented DNA was end-repaired and ligated to P1 (5′-CCACTACGCCTCCGCTTTCCTCTCTATGGGCAGTCGGTGAT-3′) and P2 (5′-CTGCCCCGGGTTCCTCATTCTCTGTGTAAGAGGCTGCTGACGGCCAAGGCG-3′) adapters that provide the primary sequences for both amplification and sequencing of the sample library fragments. The P2 adapter contains a 10-bp barcode sequence, which provided the basis for multiplex sequencing (5500 SOLiD Fragment Library Barcode Adaptors; LT). The templates were size-selected using the Agencourt AMPure XP system (Beckman Coulter), nick-translated using Platinum PCR Amplification Mix and the template library was quantitated by quantitative PCR using SOLiD Library TaqMan Quantitation Kit (LT). The templates were clonally amplified by emulsion PCR with P1 primer covalently attached to the bead surface. Emulsions were broken with butanol, and emulsion PCR beads enriched for template-positive beads by hybridization with P2-coated capture beads. Template-enriched beads were extended at the 3′ end in the presence of terminal transferase and 3′ bead linker. Beads with clonally amplified DNA were then deposited onto a SOLiD Flowchip, the slide was loaded into a SOLiD 5500xl System (LT) and the 50-base sequences were obtained according to the manufacturer’s protocol.

### Bioinformatics analysis of genome sequences

The obtained sequences from each strain were first trimmed to filter out low-quality reads that were <50 bp. The remaining high-quality sequences from each strain were then aligned to the *E. coli* K-12 substr. MG1655 chromosome (GenBank accession number NC000913; version NC_000913.2 GI:49175990) in color space using Genomics Workbench 6.0.1 (CLC Bio). An average coverage of >130-fold was accomplished for each strain. The maximum gap and mismatch count within a single read was set to 2 with a minimum of four reads to call a potential variation before further analysis. Variations represented only in the MAGE evolved lines and not in the constructed ancestrals (with the exception of mutations within the oligonucleotide target sites) were judged as off-target mutations arising during MAGE cycles.

### Mutation rate measurements

Mutation rates to rifampicin resistance were estimated using fluctuation analysis as previously described (18). Briefly, 12 tubes of 1-ml LB^L^ were inoculated with ∼10^4^ cells from each strain. Cells were grown at the examined temperature until early stationary phase. Appropriate dilutions were spread onto nonselective LB^L^ agar plates as well as LB^L^ agar plates containing rifampicin (100 µg/ml) and incubated at 30°C. Colony counts were performed after 24 or 48 h, respectively. The mutation rate was calculated using the Ma-Sandri-Sarkar maximum likelihood method (19). The calculations were performed using the FALCOR web tool (20).

### Estimating effects of temperature increase on growth parameters of MGλ-tMMR

Growth rate measurements were obtained by growing replicates of the ancestral strains, *E. coli* K-12 MG1655 wild-type, MGλ-tMMR and MGλ-Δ*mutS*. Briefly, cultures (0.6–1 µl) of the studied strains, incubated at 30°C until early stationary phase, were inoculated into 96-well shallow plates (30 replicates per strains). Each well contained 100-µl LB^L^ medium. Growth curves were recorded by measuring OD_600_ every 7 min for 24 h at the examined temperature using a Biotek-automated plate reader. Growth rate, relative to wild-type *E. coli* K-12 MG1655, was calculated from the obtained growth curves following a reported procedure (21,22).

## RESULTS

### Characterization of the temperature-sensitive MMR mutant

Using standard genome engineering techniques (see ‘Materials and Methods’ section), we introduced temperature-sensitive MMR protein alleles [MutS(A134V) and MutL(G62S)] (14) into *E. coli* K-12 MG1655 expressing the λ Red recombinases, resulting in the strain MGλ-tMMR.

**Figure 1.**
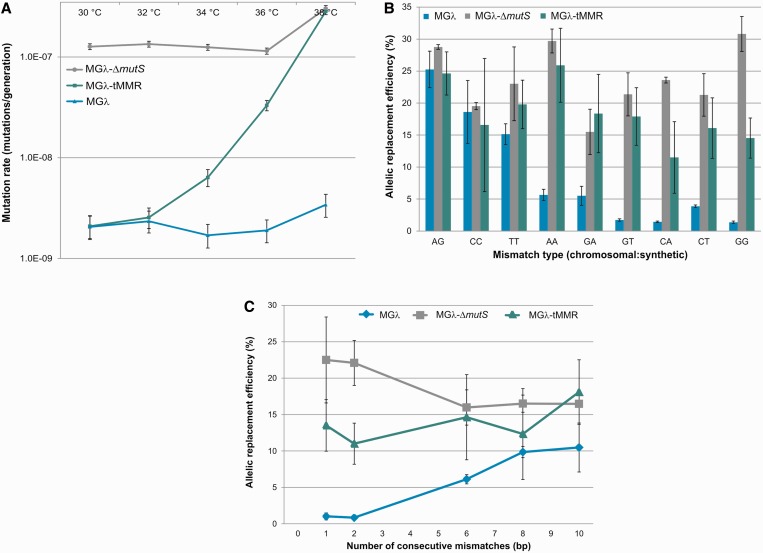
(**A**) Mutation rate measurement of employed strains at various temperatures. A rifampicin resistance assay was used to calculate mutation rates as described in ‘Materials and Methods’ section. Error bars represent 95% confidence intervals of pooled samples of two independent measurements of 12 parallel samples each. (**B**) Allelic replacement efficiencies of oligos generating various types of single base pair modifications in the chromosome, designated by chromosomal base:oligo base in MGλ, MGλ-Δ*mutS* and MGλ-tMMR. The efficiency of allelic replacement was estimated by the number of mutant cells per total colony-forming units. The values are the means of two independent measurements each, error bars represent standard errors. An A:G (G:A) mismatch was created two separate times using two different oligos. (**C**)Allelic replacement efficiencies of oligos generating modifications of increasing size in the genome of MGλ, MGλ-Δ*mutS* and MGλ-tMMR. The values are the means of two independent measurements each, error bars represent standard errors. For details, see ‘Materials and Methods’ section.

Using rifampicin resistance assay and subsequent fluctuation analysis (18), we estimated the mutation rate in this strain across a range of temperature settings. In agreement with published results (14), we observed an ∼100-fold increase in mutation rate as the temperature shifted from 32 to 38°C (Figure 1A).

To test the effect of this temperature shift (and consequent disruption of the MMR machinery) on the replacement efficiency of ssDNA oligos, we worked with a set of previously designed oligos (12). These oligos introduce all possible single base pair mismatches (A:A, C:C, G:G, T:T, G:A, G:T, C:A, C:T) at specific genomic locations, and by generating premature stop codons, they either inactivate *lacZ* or *malK*. The frequency of the corresponding allelic replacement events was thus easily detectable by colorimetric assays (see ‘Materials and Methods’ section). We found that in six of the eight cases, the MGλ-tMMR strain allowed efficient and mostly unbiased oligo incorporation comparable with that achieved by the isogenic mismatch repair knockout strain MGλ-Δ*mutS* (Figure 1B). Although the incorporation rates of C:A and G:G mismatches were significantly lower in the MGλ-tMMR strain than that in MGλ-Δ*mutS*, the observed rates were still one order of magnitude higher compared with the values seen in the MGλ strain. Additionally, we tested the allelic replacement efficiency of another set of oligos (12), which introduce mismatches of increasing lengths into *lacZ*. Again, the replacement efficiencies for 1 and 2 consecutive mismatches in both MGλ-Δ*mutS* and MGλ-tMMR strains were over an order of magnitude higher than what was observed in the MGλ strain (Figure 1C). We conclude that MGλ-tMMR and MGλ-Δ*mutS* provide similarly high allelic replacement efficiencies, both in the cases of single nucleotide changes and small insertions.

### Design and optimization of the modified protocol

**Figure 2.**
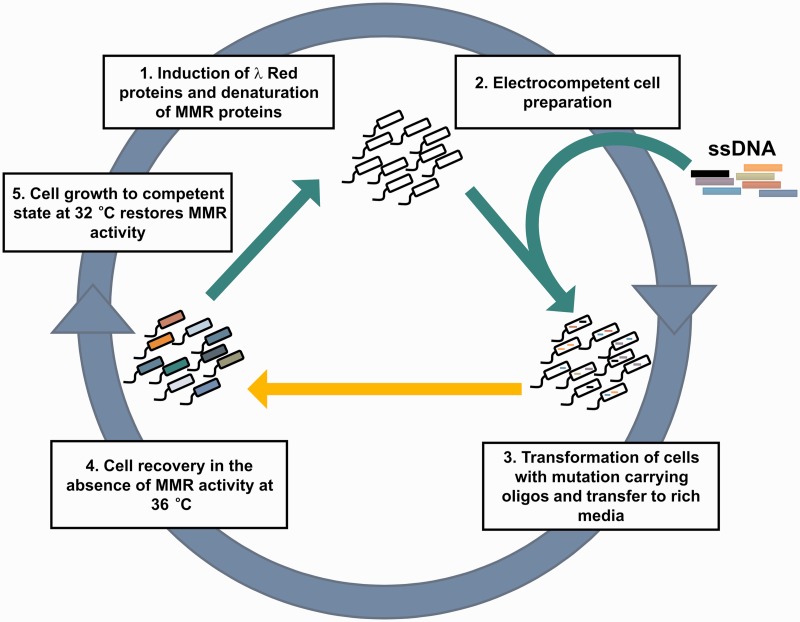
General scheme of the modified MAGE protocol. ssDNA oligos are incorporated into the bacterial genomes in a cyclical manner. The orange arrow represents the main novelty of the modified procedure, a recovery period at 36°C to which the mutator state is restricted to. See main text for details. Adapted from Wang *et al.* (2).

Based on these results, we designed a slightly modified MAGE cycle (Figure 2). Steps of the procedure are as follows: (i) a brief heat-shock (42°C, 15 min) that serves for transcriptional activation of the λ Red enzymes in the original procedure (2) and can simultaneously inactivate MMR proteins in MGλ-tMMR, (ii) electrocompetent cell preparation and (iii) transformation of the ssDNA oligos into the cells (0°C, 30 min). The λ Red Beta-mediated incorporation of the synthetic oligonucleotides into the target genome occurs during (iv) the cell recovery period (36°C, 60 min), during which the elevated temperature ensures MMR protein inactivity. This step is followed by (v) outgrowth phase at a lowered incubation temperature (32°C, 120–180 min). During the last phase, MMR protein functionality is quickly restored, and cells can replicate with low background genomic mutation rates.

**Figure 3.**
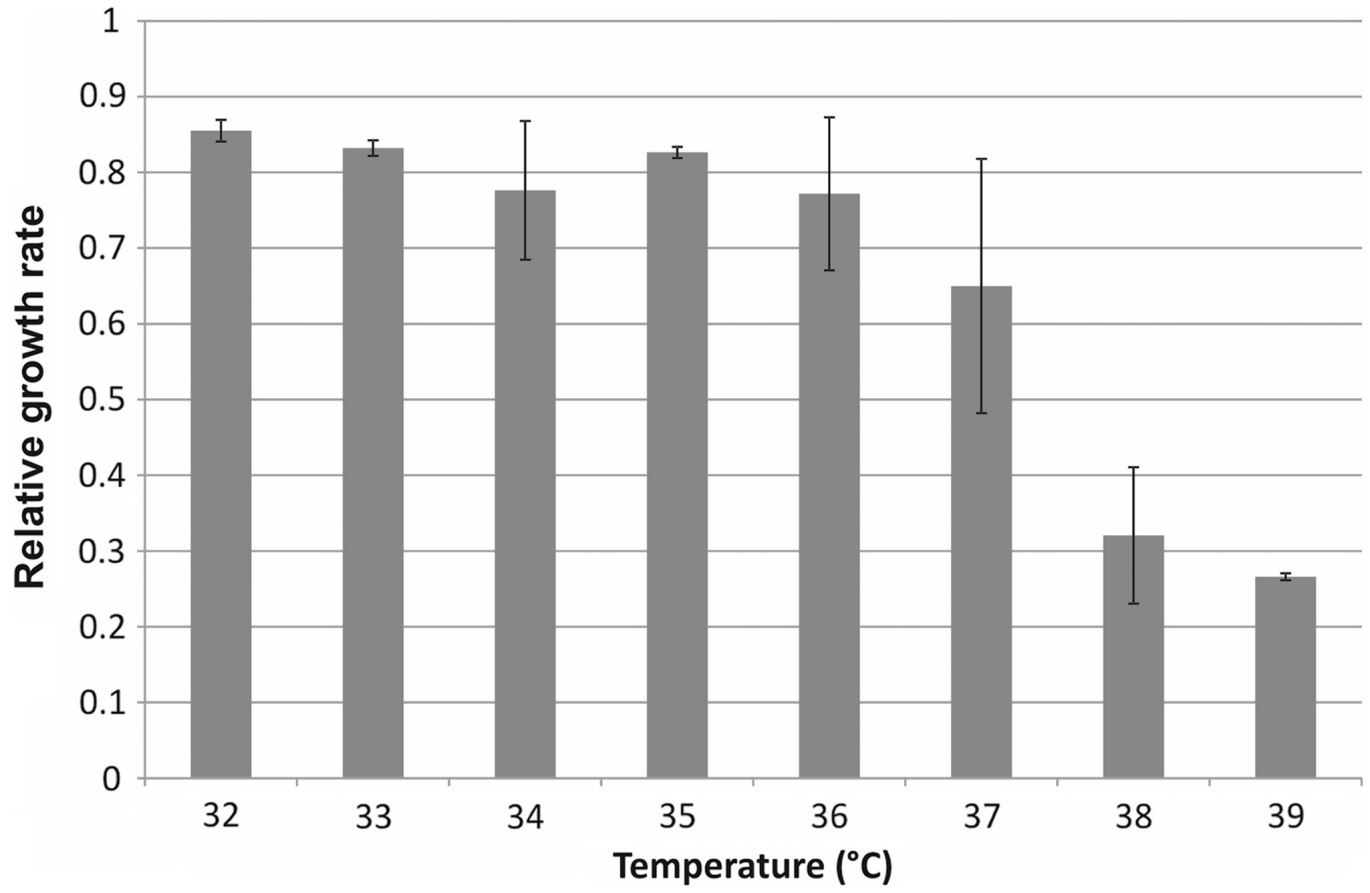
Effects of temperature increase on growth parameters of MGλ-tMMR. Relative growth rate of MGλ-tMMR, compared with wild-type *E. coli* MG1655 (wild-type = 1), at gradually elevating temperatures. Values shown are the mean of 30 replicates each, error bars represent 95% confidence intervals.

**Figure 4.**
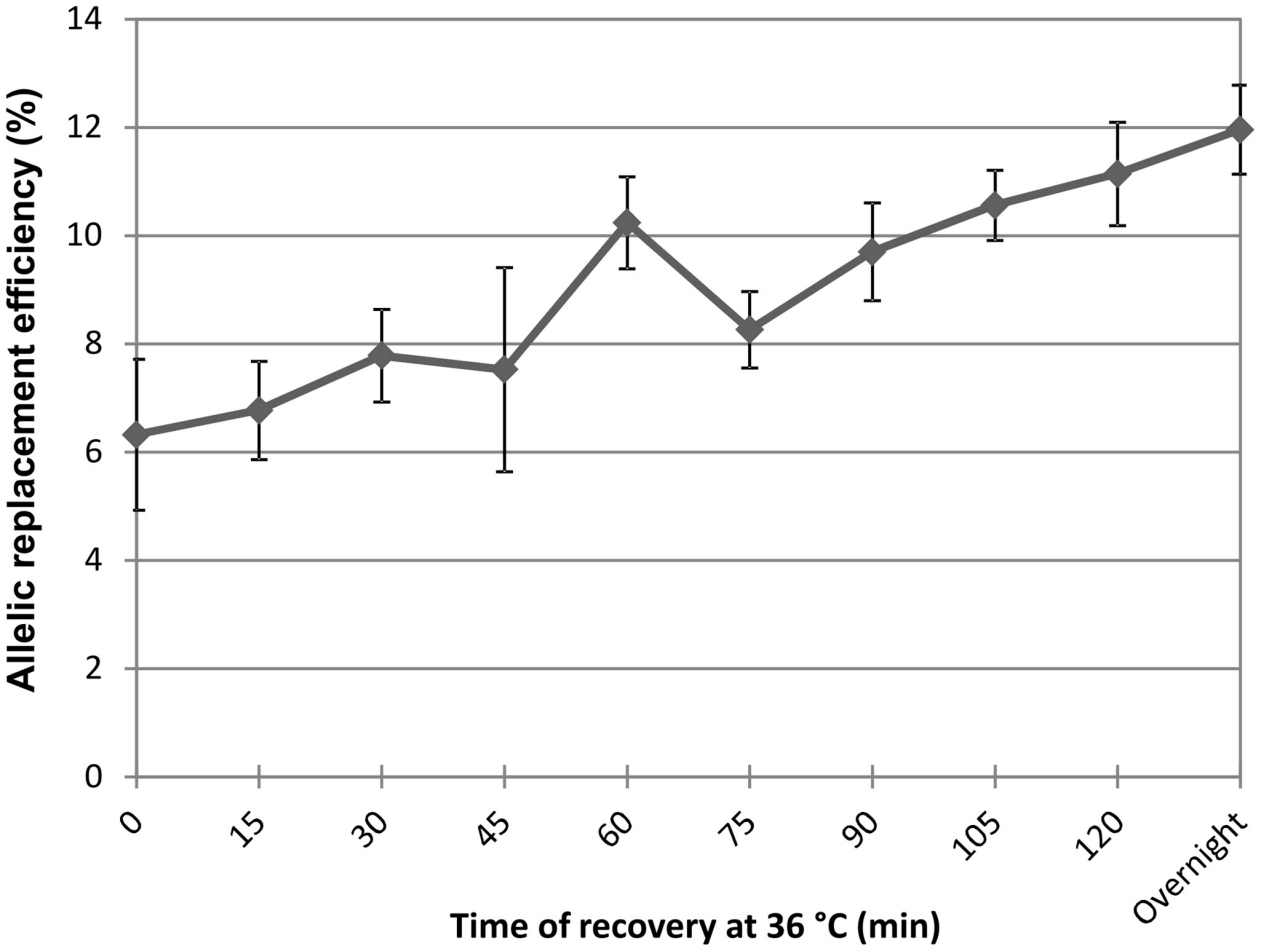
Optimization of recovery time at 36°C. Allelic replacement efficiency of a representative oligo that introduces a nonsense mutation by two consecutive mismatches in *lacZ*. Values shown are the mean of four independent experiments, error bars represent 95% confidence intervals.

The main novelty of this procedure is that the mutator state coupled to growth is restricted to a brief period (phase 4). The settings for the two crucial variables, temperature and length in phase 4 (36°C and 60 min), were determined by an optimization process. Importantly, elevated temperature induces the expression of the toxic λ p_L_ operon (23), necessary for Red Beta-mediated incorporation of synthetic oligonucleotides. Although the optimal induction temperature of this operon is at 42°C, growth retardation due to leaky expression was observed at lower temperatures as well, all the way down to 37°C (Figure 3). At 36°C, both MMR protein activity and λ p_L_ operon transcription remained sufficiently repressed, ensuring both undetectable cellular toxicity and high allelic replacement efficiency (Figure 1A). Regarding the optimal time of recovery at 36°C, the answer appears to be more complex. We tested the allele replacement efficiency of a test oligonucleotide at different recovery time lengths, and found a near linear correlation between the two variables (Figure 4). However, a longer recovery period has at least two drawbacks: it extends the length of each MAGE cycle and the amount of time the population spends in a mutator state. Sixty minutes recovery time was found to be a good compromise, as allele-replacement efficiency was sufficiently high (Figure 4), and it simultaneously minimized the chance of accumulating off-target mutations. We anticipate that depending on the exact goals of future experiments, the protocol can be optimized further, e.g. by the shortening of the recovery period (phase 4). The results indicate that the omission of phase 4 caused only a 40% decrease in the incorporation efficiency of an otherwise efficiently MMR-corrected 2-bp mismatch (Figure 4).

### Estimating the reliability of the improved MAGE protocol

To compare the performances of the original and the improved protocols, we carried out 20 cycles of MAGE using MGλ-Δ*mutS* and MGλ-tMMR populations evolving in parallel. In the case of MGλ-tMMR, the MAGE protocol was modified as described above. We used synthetic oligos that introduce various types of mismatches and target six different genes distributed widely across the genome (Supplementary Figure S1).

Allelic-replacement efficiencies for each of the six oligos were evaluated after 10 and 20 cycles of MAGE (Table 2). The high rate of allelic replacements observed in MGλ-Δ*mutS* was mostly reproduced in MGλ-tMMR, the only exceptions being the *araB* A:A and *lacZ* T:T transitions. Even though allelic replacement efficiencies after 20 cycles of these two oligos were significantly lower in the MGλ-tMMR strain, the method is still adequate in introducing these two types of mutations into the target strain at high efficiencies (see Figure 1B). Errors are known to occur during allelic replacement due in part to defects arising from oligo synthesis (5). To investigate whether this had a different effect in the two protocols, we sequenced one of the targeted genes (*cycA*) in 96 isolated parallel evolved clones after 20 cycles from both strains. From MGλ-tMMR, 6 of 96 sequenced clones had within-site mutations in regions corresponding to the annealing site of the 90-mer MAGE oligo, cycA_AAAC, whereas 9 of 96 clones from MGλ-Δ*mutS* carried such mutations. The difference in the number of erroneous replacements is not significant [*X*^2^ (2, N = 192) = 0.65, *P* = 0.42]. The principal errors in the MGλ-Δ*mutS* clones were single base deletions. The majority of mutations observed in MGλ-tMMR were nucleotide changes, but 4 of 5 originated from a specific change of C→T. This is most likely linked to synthesis error during oligonucleotide production (data not shown). We conclude that the two constructs exhibit similarly low error rates and high efficiency of allelic replacements.

**Table 2.** Allelic replacement efficiencies of all six used oligos in MGλ-Δ*mutS* and MGλ-tMMR after 10 and 20 MAGE cycles

Allelic replacement ratio (%)				
Gene (mismatch type) (chromosomal base: oligo base)	10 cycle population		20 cycle population	
	MGλ-Δ*mutS*	MGλ-tMMR	MGλ-Δ*mutS*	MGλ-tMMR
malK (C:C)	52.08	38.54	68.75	71.88
cycA (AA:AC)	55.20	47.91	85.42	88.54
araB (A:A)	40.62	23.95*	58.33	29.17**
lacZ (T:T)	72.91	66.66	94.79	71.88**
hisB (G:T)	59.37	62.50	89.58	89.58
rpsL (A:C)	60.41	63.54	85.42	76.04

Fisher’s exact test was performed comparing each value of the MGλ-tMMR strain with the corresponding value of the MGλ-Δ*mutS* strain, *P < 0.05, **P < 0.01.

Next, we investigated the accumulation of off-target mutations. After 20 cycles of MAGE, we selected five independently evolved clones derived from both MGλ-tMMR and MGλ-Δ*mutS*. All of them were verified to carry all six specific targeted allele replacements. To infer the number of off-target mutations, the genomes of these clones were sequenced using the SOLiD 5500xl System. The sequenced clones derived from MGλ-Δ*mutS* carried 97 off-target mutations (single-nucleotide polymorphisms and indels) affecting 76 protein-coding genes (on average 19.4 point mutations per clone). In contrast, the clones derived from MGλ-tMMR accumulated only 15 mutations (3 mutations per clone). Based on these figures, we estimate that the improved MAGE protocol reduces the number of off-target mutations by ∼85%. As expected (24), most of the extra mutations in MGλ-ΔmutS-derived clones were A:T→G:C and G:C→A:T transitions, and they were nearly completely absent in MGλ-tMMR-evolved clones (Table 3). For a full list of all off-target mutations, see Supplementary Table S1.

**Table 3.** Number and types of off-target mutations observed in the genomes of MGλ-Δ*mutS* and MGλ-tMMR-derived strains after 20 MAGE cycles

Types of off-target mutations				
	MGλ-Δ*mutS*		MGλ-tMMR	
	Number	Fraction	Number	Fraction
Transitions				
A:T > G:C	34	35.1	3	20
G:C > A:T	52	53.6	9	60
Transversions				
A:T > T:A	0	0	1	6.7
A:T > C:G	0	0	0	0
G:C > T:A	0	0	1	6.7
G:C > C:G	0	0	0	0
Number of insertions	8	8.2	1	6.7
Number of deletions	3	3.1	0	0
Total	97		15	
Consequences of substitutions				
Position				
Noncoding	14	14.4	2	13.3
Coding	83	85.6	13	86.7
Within coding sequences				
Synonymous	21	25.3	1	7.7
Nonsynonymous	62	74.7	12	92.3

## DISCUSSION

Techniques based on oligonucleotide-mediated allelic replacement have several properties that make them especially promising for genome-scale engineering projects (25). Allelic replacement is a general mechanism that allows for the targeted modification of genes without the need for restriction endonuclease sites, antibiotic selection of integrated constructs, or the generation of unwanted ‘scars’ in the vicinity of the target modification. Additionally, the high transformation efficiency and low cost of single-stranded DNA oligos allow for the simultaneous modification of multiple targets within a single cell. The MAGE method has been used to achieve a number of key innovations, including the generation of a so-called genomically recoded organism, constructed by the genome-wide replacement of a specific codon (6). However, this recent breakthrough also pointed out the major drawback of the technique. Besides the targeted 321 modifications, 355 unwanted off-target mutations were detected, owing to the applied DNA repair-deficient cellular background (6). These undesired mutations caused a reduction in fitness in the engineered strains. Correction of particularly disadvantageous off-target mutations is a painstaking approach that presents the added possibility of generating additional off-target mutations. Therefore, it is apparent that future genome engineering endeavors will need a more precise *in vivo* genome-editing approach with reduced off-target effects to achieve increased genome stability.

In this work, we described a novel strategy with an aim to concurrently maintain low genomic mutation rate and high allelic replacement efficiency during multiplex genome engineering. We used temperature-sensitive MMR protein variants to decrease background mutations by temporal inactivation of DNA repair activity during genome editing. Compared with the traditional workflow, our protocol decreased the number of off-target mutations by 85%. Importantly, this result was achieved without significantly decreasing the efficacy and accuracy of oligo-mediated allelic replacement, or the speed of the MAGE cycles. Moreover, the protocol is simple and can be easily automated in the laboratory: it only requires a shift in incubation temperature during cell recovery and the use of a modified strain. Furthermore, our general guideline of temporal inactivation of MMR may be applicable to genome engineering of other organisms (26–31). Notably, transient downregulation of MMR proteins using RNA interference to allow for oligonucleotide-mediated gene targeting has been demonstrated in murine embryonic stem cells (32,33). Importantly, oligonucleotide-mediated recombination engineering has recently been adapted for yeast (*Saccharomyces cerevisiae*) (34), and mismatch repair protein variants with temperature-sensitive defects have been found in the same species (35). In summary, our work improves the precision of oligonucleotide-mediated genome engineering, thereby allowing for more predictable and highly efficient cell programming for synthetic biological and industrial applications.

## SUPPLEMENTARY DATA

Supplementary Data are available at NAR Online.

## FUNDING

European Research Council (to C.P.); Wellcome Trust (to C.P.); Lendület Program of the Hungarian Academy of Sciences (to C.P.); Hungarian Research Fund [OTKA PD 109572 to B.C.]. Funding for open access charge: Wellcome Trust.

*Conflict of interest statement*. None declared.

## Supplementary Material

Supplementary Data
