# Supplementary material for: Conditional DNA repair mutants enable highly precise genome engineering
Source: Nucleic Acids Res. 2014 Feb 5;42(8):e62. doi: 10.1093/nar/gku105 (PMC4005651; doi:10.1093/nar/gku105)
Supplement: Supplementary Data [file supp_42_8_e62__index.html]

Conditional DNA repair mutants enable highly precise genome engineering — Conditional DNA repair mutants enable highly precise genome engineering — Supplementary Data 

# Conditional DNA repair mutants enable highly precise genome engineering

## Supplementary Data

files

**Files in this Data Supplement:**

- Supplementary Data - pdf file
- Supplementary Data - xlsx file
