# Supplementary material for: Conditional DNA repair mutants enable highly precise genome engineering
Source: Nucleic Acids Res. 2014 Feb 5;42(8):e62. doi: 10.1093/nar/gku105 (PMC4005651; doi:10.1093/nar/gku105)
Supplement: Supplementary Data [file supp_gku105_nar-03457-met-f-2013-File011.pdf]

Supplementary Figure 1**.**

Genomic map and positions of the 6 targeted genes and introduced modifications (mismatch marked as nucleotide mispair, in chromosomal to synthetic order) across the *E. coli* K-12 substr. MG1655 chromosome. The choice of these target genes was based on the following criteria: First, previously published markers (3, 12) should be easily detectable (ensuring easy detection of gene modification). Second, the genes should be more or less uniformly distributed on the bacterial chromosome (to minimize any potential biases from regional variation of mutation rate across the genome). Third, to ensure rigorous testing of allele replacement efficiency, the introduced, selectable modifications should cover various base substitutions and consecutive mismatches.

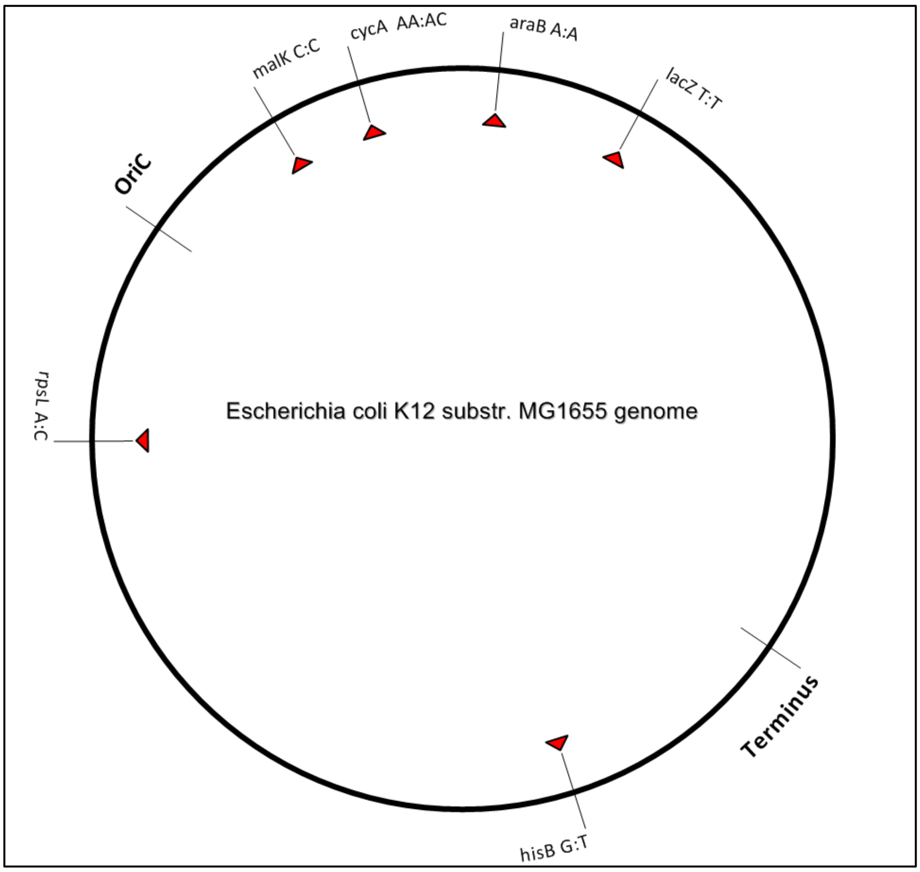

Gene	Genomic position	Mismatch	Corresponding oligo
araB	69999	A:A	araB_AA
lacZ	364878	T:T	LacZ_TT_v7
hisB	2091657	G:T	hisB_GT
rpsL	3472447	A:C	rpsL_AC
malK	4245058	C:C	MalK_CC_v1
cycA	4428025 - 4428026	AA:AC	cycA_AAAC

Supplementary Table 1.

List of all off-target mutations observed following 20 cycles of MAGE.

Strain	Reference nucleotide position	Variation type	Wild type	Mutant	Annotations	Coding region change	Amino acid change
dMUTS1	198053	SNV	C	T	Gene: bamA, CDS: bamA, Mature peptide	126C>T	
	500989	SNV	C	T	Gene: ybaL, CDS: ybaL	1474G>A	Gly492Ser
	809211	SNV	G	A	Gene: bioB, CDS: bioB	645G>A	
	856275	SNV	A	G	Gene: ybiT, CDS: ybiT	1090A>G	Thr364Ala
	1266980	SNV	G	A	Gene: ychA, CDS: ychA	438G>A	
	1511304	SNV	T	C	Gene: ydcT, CDS: ydcT	464T>C	Val155Ala
	1723026	SNV	A	G	Gene: ydhF, CDS: ydhF	631T>C	Cys211Arg
	1969189	SNV	T	C	Gene: tar, CDS: tar	1527A>G	
	2177201	SNV	T	C	Gene: yegT, CDS: yegT	359T>C	Val120Ala
	2357301	SNV	T	C	Gene: rhmT, CDS: rhmT	874A>G	Thr292Ala
	2481492	Deletion	A	-			
	3090053	SNV	G	A	Gene: gshB, CDS: gshB	154G>A	Ala52Thr
	3454941	SNV	A	G	Gene: gspD, CDS: gspD	543A>G	Ile181Met
	3534326	SNV	C	T	Gene: ompR, CDS: ompR	281G>A	Gly94Asp
	3535473	SNV	G	A	Gene: yhgF, CDS: yhgF	67G>A	Ala23Thr
	3844792	SNV	G	A	Gene: uhpT, CDS: uhpT	399C>T	
	4190408	SNV	A	G	Gene: thiG, CDS: thiG	251T>C	Leu84Pro
	1707433	Insertion	-	C	Gene: rsxD, CDS: rsxD	267_268insC	Pro90 fs
dMUTS2	516333	SNV	A	G	Gene: ybbM, CDS: ybbM	527A>G	Asp176Gly
	1099170	SNV	C	T	Gene: ycdY, CDS: ycdY	308C>T	Ala103Val
	1141087	SNV	G	A	Gene: rne, CDS: rne	2504C>T	Pro835Leu
	1152009	SNV	C	T	Gene: fabF, CDS: fabF	848C>T	Ala283Val
	1211304	Insertion	-	C			
	1348135	SNV	C	T			
	1463917	SNV	G	A	Gene: ydbA, CDS: ydbA	502G>A	Ala168Thr
	1816568	Deletion	C	-			
	2142854	SNV	G	A	Gene: yegE, CDS: yegE	1565G>A	Arg522Gln
	2273825	SNV	A	G	Gene: yejE, CDS: yejE	531A>G	
	3092810	SNV	G	A	Gene: yggR, CDS: yggR	293C>T	Ser98Leu
	3248018	SNV	G	A	Gene: yqjE, CDS: yqjE	314G>A	Arg105His
	3534292	Insertion	-	T	Gene: ompR, CDS: ompR	315_316insA	Pro106 fs
	3968050	Insertion	-	G	Gene: wzzE, CDS: wzzE	996_997insG	Gly333 fs
dMUTS3	72811	SNV	A	G	Gene: thiQ, CDS: thiQ	117T>C	
	337498	SNV	C	T	Gene: yahF, CDS: yahF	1497C>T	
	473782	SNV	C	T	Gene: tesB, CDS: tesB, Mature peptide	604G>A	Ala202Thr
	839090	SNV	A	G	Gene: fiu, CDS: fiu	1665T>C	
	1526035	SNV	C	T	Gene: rhsE, CDS: rhsE	110C>T	Thr37Met
	1699258	SNV	G	A	Gene: malY, CDS: malY	278G>A	Gly93Asp
	1705146	SNV	C	T	Gene: rsxC, CDS: rsxC	204C>T	
	2055694	Insertion	-	C			
	2332434	SNV	T	C	Gene: yfaT, CDS: yfaT	548A>G	Lys183Arg
	2697672	SNV	T	C			

	2938371	SNV	G	A	Gene: rlmM, CDS: rlmM	895C>T	Arg299Cys
	3132618	SNV	C	T	Gene: yghT, CDS: yghT	466C>T	His156Tyr
	3212090	SNV	T	C	Gene: rpoD, CDS: rpoD	1022T>C	Leu341Pro
	3392101	SNV	T	C	Gene: yhdP, CDS: yhdP	2180A>G	Asn727Ser
	3482189	SNV	G	A	Gene: yheT, CDS: yheT	966G>A	Trp322 STOP
	3534246	SNV	G	A	Gene: ompR, CDS: ompR	361C>T	Arg121Cys
	3571638	SNV	C	T			
	3805846	SNV	T	C	Gene: rfaQ, CDS: rfaQ	276A>G	
	3957957	SNV	C	T			
	4285209	Insertion	-	G	Gene: acs, CDS: acs	186_187insC	Gly63 fs
	4350904	SNV	C	T			
	4516166	SNV	T	C	Gene: fecI, CDS: fecI	93A>G	
dMUTS4	133663	SNV	T	C	Gene: acnB, CDS: acnB	2049T>C	
	808413	Insertion	-	G	Gene: bioA, CDS: bioA	68_69insC	Pro23 fs
	1046492	SNV	C	T	Gene: gfcD, CDS: gfcD	677G>A	Gly226Glu
	1434260	SNV	A	G	Gene: ompN, CDS: ompN, Mature peptide	658T>C	Ser220Pro
	1511304	SNV	T	C	Gene: ydcT, CDS: ydcT	464T>C	Val155Ala
	1535969	SNV	G	A	Gene: narY, CDS: narY	909C>T	
	2385471	SNV	G	A			
	2584224	SNV	G	A	Gene: narQ, CDS: narQ	472G>A	Gly158Ser
	2764644	SNV	T	C	Gene: rnlA, CDS: rnlA, cryptic prophage CP4-57	705T>C	
	3143698	SNV	G	A	Gene: hybO, CDS: hybO, Mature peptide	586C>T	His196Tyr
	3478236	SNV	A	G	Gene: kefB, CDS: kefB	394T>C	
	3534326	SNV	C	T	Gene: ompR, CDS: ompR	281G>A	Gly94Asp
	3949063	SNV	A	G	Gene: ilvG, CDS: ilvG	481A>G	Ile161Val
	3957957	SNV	C	T			
	4531516	SNV	C	T	Gene: yjhQ, CDS: yjhQ	292G>A	Gly98Arg
dMUTS5	297040	SNV	A	G	Gene: paoD, CDS: paoD	911T>C	Val304Ala
	297847	SNV	A	G	Gene: paoD, CDS: paoD	104T>C	Leu35Pro
	967391	SNV	A	G	Gene: msbA, CDS: msbA	1548A>G	
	991336	SNV	C	T	Gene: pepN, CDS: pepN	1492C>T	Pro498Ser
	1039457	SNV	C	T	Gene: appB, CDS: appB	939C>T	
	1258131	SNV	C	T	Gene: ychH, CDS: ychH	118C>T	Gln40 STOP
	1303942	SNV	C	T	Gene: oppF, CDS: oppF	155C>T	Thr52Ile
	1338764	SNV	G	A	Gene: yciM, CDS: yciM	183G>A	Met61Ile
	1368919	SNV	G	A	Gene: ycjM, CDS: ycjM	680G>A	Arg227His
	1528263	SNV	A	G	Gene: ydcD, CDS: ydcD	318A>G	
	1538289	SNV	A	G	Gene: narZ, CDS: narZ	2326T>C	Ser776Pro
	2071792	SNV	G	A	Gene: flu, CDS: flu, cryptic prophage CP4-44	2230G>A	Val744Ile
	2090357	SNV	G	A	Gene: hisD, CDS: hisD, Mature peptide	1237G>A	Ala413Thr
	2765461	Insertion	-	C	Cryptic prophage CP4-57		
	2841559	SNV	T	C	Gene: hycG, CDS: hycG	674A>G	Gln225Arg
	2867747	SNV	G	A	Gene: surE, CDS: surE	550C>T	Gln184 STOP
	2930610	SNV	A	G	Gene: fucO, CDS: fucO	426T>C	
	3021284	SNV	C	T	Gene: xdhD, CDS: xdhD	1947C>T	
	3284177	Deletion	G	-	Gene: agaD, CDS: agaD	678delG	Met226 fs
	3390059	SNV	C	T			
	3395171	SNV	G	A	Gene: rng, CDS: rng, Mature peptide	647C>T	Ala216Val

	3534338	SNV	T	C	Gene: ompR, CDS: ompR	269A>G	Asp90Gly
	3957957	SNV	C	T			
	3987065	SNV	C	T	Gene: hemX, CDS: hemX	25G>A	Ala9Thr
	4233215	SNV	C	T	Gene: pgi, CDS: pgi	1435C>T	Pro479Ser
	4405349	SNV	G	A	Gene: rnr, CDS: rnr, Mature peptide	673G>A	Asp225Asn
	4480647	SNV	A	G	Gene: valS, CDS: valS	1214T>C	Val405Ala
	4599861	SNV	A	G	Gene: yjjB, CDS: yjjB	260T>C	Val87Ala
tMMR1	3990080	SNV	A	G	Gene: cyaA, CDS: cyaA	905A>G	Tyr302Cys
tMMR2	389627	SNV	A	T	Gene: yaiT, CDS: yaiT	153A>T	
	3533981	SNV	C	T	Gene: ompR, CDS: ompR	626G>A	Arg209His
tMMR3	287981	SNV	C	T	Gene: yagI, CDS: yagI, cryptic prophage CP4-6	406G>A	Ala136Thr
	1962479	SNV	T	C	Gene: flhA, CDS: flhA	596A>G	Asp199Gly
	3533981	SNV	C	T	Gene: ompR, CDS: ompR	626G>A	Arg209His
	3957957	SNV	C	T			
tMMR4	1963018	SNV	C	T	Gene: flhA, CDS: flhA	57G>A	Trp19 STOP
	1986454	SNV	T	C	Gene: yecR, CDS: yecR	209T>C	Val70Ala
	3533981	SNV	C	T	Gene: ompR, CDS: ompR	626G>A	Arg209His
	3957957	SNV	C	T			
tMMR5	3532875	SNV	C	A	Gene: envZ, CDS: envZ	1016G>T	Arg339Leu
	3707579	Insertion	-	C	Gene: eptB, CDS: eptB	920_921insG	Gly307 fs
	3800849	SNV	G	A	Gene: rfaI, CDS: rfaI	233C>T	Ala78Val
	3989698	SNV	G	A	Gene: cyaA, CDS: cyaA	523G>A	Gly175Ser
